# Development of Next Generation Probiotics for Cardiometabolic Diseases

**DOI:** 10.1007/s43657-025-00230-z

**Published:** 2025-03-21

**Authors:** Muhammed Tanweer Khan, Fredrik Bäckhed

**Affiliations:** 1 Wallenberg Laboratory, Department of Molecular and Clinical Medicine, Institute of Medicine, University of Gothenburg, Gothenburg, Sweden 41345; 2 Biogaia AB, 112 27 Stockholm, Sweden; 3 Department of Clinical Physiology, Region Västra Götaland, Sahlgrenska University Hospital, Gothenburg, Sweden 41345

**Keywords:** Probiotics, Cardiometabolic disease, Gut microbiota

The adult human gut microbiota consists of at least as many bacterial cells as our total number of somatic and germ cells (Sender et al. 2016). Their collective genomes (microbiome) contain 2–3 order of magnitude more genes (Li et al. 2014) and can accordingly complement our own human genome with functions we did not need to develop. The gut microbiota was clearly demonstrated to protect and even treat *Clostridioides difficile* (earlier known as *Clostridium difficile*) infections in humans (van Nood et al. 2013), clearly demonstrating the importance of an intact microbiota for maintaining health.

Numerous metagenomic studies have revealed that microbiomes associated with different diseases are characterized with reduced microbial diversity and reduced abundance of butyrate producing bacteria, such as members of *Roseburia* and the anti-inflammatory *Faecalibacterium prausnitzii* (Allin et al. 2018; Cao et al. 2014; Qin et al. 2012; Sokol et al. 2008; Wu et al. 2020). These taxa are highly discriminant for type 2 diabetes (T2D) and have been linked to improved insulin sensitivity (Karlsson et al. 2013) and fecal microbiota transplantations from lean donors to recipients with metabolic syndrome improved insulin sensitivity (Vrieze et al. 2012). Accordingly, we also demonstrated that *F. prausnitzii* is the most discriminative taxa comparing individuals with prediabetes and diabetes-treatment naïve individuals with healthy controls (Wu et al. 2020), suggesting the importance of butyrate-producing bacteria for blood glucose regulation in humans. Dietary fibers are the classical substrate for butyrate production and accordingly the gut microbiota protects against atherosclerosis development in *Apoe-/-* mice in the presence of fiber (Lindskog Jonsson et al. 2018). To further investigate the specific interaction between fibers and butyrate producing bacteria, germ-free mice were colonized with a consortium of eight bacteria in the absence and presence of the butyrate producing bacteria *R. intestinalis* (Kasahara et al. 2018). The mice were protected against atherogenesis when mice were fed a polysaccharide-rich diet, but not the control diet. However, fermentation is the major energy producing process for the gut microbiota and disposal of fermentation electron-sink by-products such as lactate and hydrogen is essential to maintain fermentative processes (Wang et al. 2020). Accordingly, hydrogen scavengers such as methanogens and sulfate reducing bacteria are also important for establishing gut metabolic networks (Smith et al. 2019). Removal of hydrogen from the gut ecosystem potentially increases fermentation capacity, leading to higher energy gain from the diet and increased production of short chain fatty acids (Turnbaugh et al. 2006), while methane can regulate gut motility (Xiao et al. 2015). Sulfate reducing bacteria, such as *Desulfovibrio piger,* remove hydrogen by obtaining sulfate from the host or diet, for example via cross-feeding facilitated by *Bacteroides*-encoded sulfatases, and hydrogen from H_2_-producing Actinobacterium *Collinsella aerofaciens* to produce acetate and hydrogen sulfide without compromising gut barrier integrity (Rey et al. 2013). In addition to T2D, *F. prausnitzii* is also relatively depleted in several diseases including inflammatory bowel disease (IBD) (Sokol et al. 2008). Targeting the gut microbiome has thus been proposed as a target for disease modulation and treatment. Accordingly, there is increasing interest in isolating bacteria that are depleted from the microbiome during specific conditions and when administered in adequate amounts to confer a health benefit are considered as next generation probiotics (O’Toole et al. 2017). In an effort to isolate sulfate reducing bacteria we serendipitously co-isolated *F. prausnitzii* with *D. piger* and could demonstrate that they had complementary metabolism that resulted in increased biomass of *F. prausnitzii* and increased butyrate production in vitro (Khan et al. 2023). Next, we demonstrated that the two bacteria were safe and well tolerated, first in mice and thereafter in healthy humans. Since the human participants were healthy and had a high abundance of *F. prausnitzii* (3.4% and 25.9%; mean 13.2%), we could not detect increased levels after treatment and it is currently not evident that increasing the levels of these bacteria would have major physiologic impact. In contrast, we found increased levels of *D. piger*, which also was associated with increased butyrate producing potential (Khan et al. 2023). This implies that administration of the two bacteria may have significant effects on gut microbiota composition. It also highlights that successful future products may require multiple strains for optimal effect.

Another example of a potential candidate for next-generation probiotics is *Christensenella minuta,* which belongs to the Christensenellaceae family and was first described in reference databases in 2012. It is associated with leanness (Goodrich et al. 2014) and healthy aging (Biagi et al. 2016). Furthermore, over the past decade, *C. minuta* and the Christensenellaceae have been shown to be depleted in metabolic and gastrointestinal disorders including obesity, hypertension, prediabetes, diabetic retinopathy, atherogenic dyslipidemia, and IBD (Akbuğa-Schön et al. 2024; Goodrich et al. 2014; He et al. 2018; Relizani et al. 2022; Sowah et al. 2022). Several other bacteria have also been indicated to beneficially modulate host metabolism including *Akkermansia muciniphila* (Depommier et al. 2019) and *Anaerobutyricum soehngenii* (Gilijamse et al. 2020). Both are depleted in patients with the metabolic syndrome and when administered to mice they improve metabolic features (Everard et al. 2013; Udayappan et al. 2016). Furthermore, both strains are safe for human consumption and preliminary data shows positive effects on glucose metabolism in mice and humans (Depommier et al. 2019; Gilijamse et al. 2020; Perraudeau et al. 2020). Interestingly, a combination of *A. muciniphila*, *A. soehngenii, Bifidobacterium infantis, C. beijerinckii*, and *C. butyricum*, together with inulin significantly improved glucose metabolism in patients with T2D (Perraudeau et al. 2020). Furthermore, a collection of 17 spore forming bacteria has obtained approval for treating *C. difficile* infections in humans (Feuerstadt et al. 2022). Accordingly, these results suggest that future products to treat cardiometabolic diseases may require several strains.

As shown in Fig. 1, there are several challenges with development of next generation probiotics, including engraftment and colonization of the gut. This is particularly important if the bacterial metabolism is required for the beneficial effects. For example, it appears as *B. infantis* strains isolated from Bangladeshi children have improved capacity to colonize children from Bangladesh (Barratt et al. 2022), which may suggest that geography and ethnicity can be important factors to consider when isolating strains for probiotic development. Another challenging factor may be to develop formulations with sufficiently high dose for observing clinical effects. For example, in the study by Khan et al. (2023). we only observed increased abundance of *D. piger* in the highest dose. Thus, it will be essential to improve yield and loss of viability during storage. One such example can be to improve the strains capacity to tolerate oxygen.

**Fig. 1:**
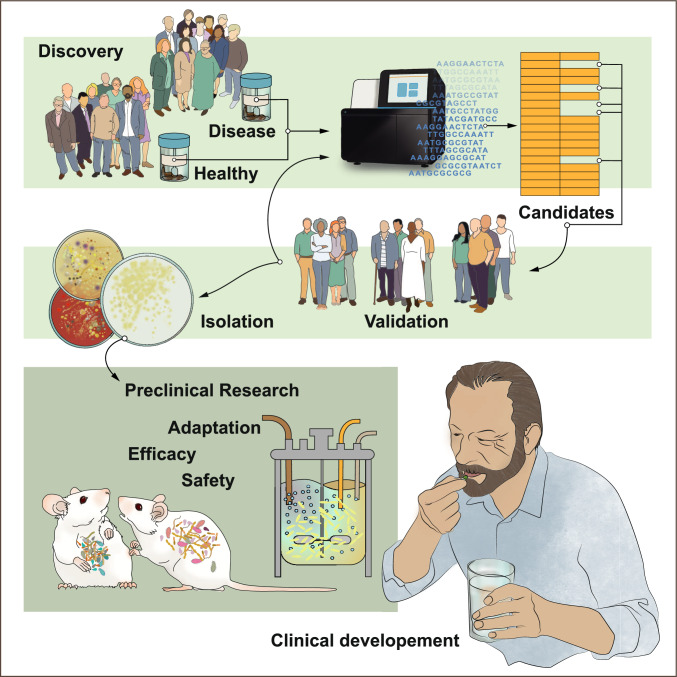
Strategy for isolation and development of next generation probiotics*.* Comparative metagenomic studies identifies bacterial strains that are depleted in disease. These candidates are then validated in additional cohorts before targeted isolation of the corresponding live organism is initiated using several substrates. Target strains thereafter need to be sequenced and phenotyped to confirm absence of virulence factors as well as to assess antibiotic resistance genes. Since many of these strains are strict anaerobes, the isolation and cultivation are performed using anaerobic chambers or gas jars. Animal models such as mice are used to assess safety before process and formulation development are initiated including "training" of the bacteria as well as upscaling to generate sufficient biomass for safety clinical trials. Process development includes the upstream process, such as the selection and optimization of growth media and conditions, as well as growth kinetics, while the downstream process focuses on concentration, cryoprotectant optimization, and freeze-drying conditions. After optimizing and validating the process, the dosage formulation is developed, which may include capsules, lozenges, or sachets

We addressed the challenge of adapting the oxygen-sensitive bacterium *F. prausnitzii* for large-scale production by cultivating it in a bioreactor designed to mimic gut conditions. The oxygen exposure was incrementally increased whereafter strains capable of surviving elevated oxygen levels were selected and isolated. This approach yielded an oxygen-tolerant *F. prausnitzii* strain that retained its capacity to produce butyrate in co-culture with *D. piger*, though the underlying molecular mechanisms remain unclear. Enhanced tolerance to abiotic stress facilitated the scale-up of *F. prausnitzii* production, including critical down-stream processes such as freeze-drying and storage stabilization, enabling the development of a formulation suitable for clinical trials (Khan et al. 2023).

Targeting the gut microbiota holds great potential for improving human health, and metagenomics studies in the last two decades have identified a broad range of bacteria that might be candidates for development of next-generation probiotics (O'Toole et al. 2017). It is important to acknowledge that the vast majority of bacteria identified in metagenomics surveys lacks cultured representatives (Almeida et al. 2021), which highlights the importance of isolation of bacteria that can be further investigated for their potential in improving host health. Further development of animal models may be used for exploring efficacy and mechanisms. Once a potential bacterial strain has been developed it will be essential to determine how to optimize both the production, for example through optimizing oxygen tolerance, but also by supplying specific fibers and/or micronutrients and vitamins that may improve the function of the bacteria (Fig. 1).

## Data Availability

Not applicable.
